# *P-V-T* equation of state of boron carbide

**DOI:** 10.1098/rsta.2022.0331

**Published:** 2023-10-16

**Authors:** Maddury Somayazulu, Muhtar Ahart, Yue Meng, Jennifer Ciezak, Nenad Velisavlevic, Russell J. Hemley

**Affiliations:** ^1^ HPCAT, X-ray Science Division, Argonne National Laboratory, Argonne, IL, USA; ^2^ Department of Physics, University of Illinois Chicago, IL, USA; ^3^ Department of Chemistry, University of Illinois Chicago, IL, USA; ^4^ Department of Earth and Environmental Sciences, University of Illinois Chicago, IL, USA; ^5^ Army Research Laboratory, Aberdeen Proving Grounds, Aberdeen, MD, USA; ^6^ Physics Division, Lawrence Livermore National Laboratory, Livermore, CA, USA

**Keywords:** P-V-T equation of state, boron carbide, synchrotron X-ray diffraction, laser heating

## Abstract

We report the *P-V-T* equation of state measurements of B_4_C to 50 GPa and approximately 2500 K in laser-heated diamond anvil cells. We obtain an ambient temperature, third-order Birch–Murnaghan fit to the *P-V* data that yields a bulk modulus *K*_0_ of 221(2) GPa and derivative, (d*K*/d*P*)_0_ of 3.3(1). These were used in fits with both a Mie–Grüneisen–Debye model and a temperature-dependent, Birch–Murnaghan equation of state that includes thermal pressure estimated by thermal expansion (*α*) and a temperature-dependent bulk modulus (d*K*_0_/d*T*). The ambient pressure thermal expansion coefficient (*α*_0 _*+ α*_1_*T*), Grüneisen *γ*(*V*) = *γ*_0_(*V*/*V*_0_)*^q^* and volume-dependent Debye temperature, were used as input parameters for these fits and found to be sufficient to describe the data in the whole *P-T* range of this study.

This article is part of the theme issue ‘Exploring the length scales, timescales and chemistry of challenging materials (Part 1)’.

## 1. Introduction

Boron carbide is a refractory, lightweight, super hard material with outstanding hardness (Vickers hardness approx. 3770 kg mm^−2^, surpassed only by diamond and cubic boron nitride), high-temperature stability (melting point greater than 2700 K at ambient pressures) and higher Hugoniot elastic limit than any other ceramic material by at least a factor of 2 (approx. 20 GPa) [1,2]. The low density and specific gravity make it ideal for use as lightweight armour and for space applications where protection from space debris impacts and resistance to radiation are both prerequisites in addition to maximizing the load–fuel ratio. Boron carbide is also used as a control material in thermal and fast reactors [3] and supercapacitor material [4]. Boron carbide is, however, shown to exhibit enigmatic behaviour under static and dynamic pressures that suggest the possibility of an elastic anomaly and loss of shear strength above 20 GPa. This behaviour complicates the application potential of B_4_C especially as a lightweight armour material [1,5].

This brittle failure of B_4_C under impact has been widely documented from shockwave experiments [2,5–7]. Apparent differences between static and dynamic high pressure experiments were resolved when it was reported that B_4_C compressed non-hydrostatically in diamond anvil cells (DACs), exhibit narrow amorphous bands (less than 10 nm in diameter) that could be identified using Raman spectroscopy and electron microscopy of the recovered samples [7,8]. Neutron and single crystal synchrotron, X-ray diffraction studies on B_4_C have alluded to the structural origin of this to be due to a ‘molecular inversion’ around 10 GPa that collapses the B_6_C icosahedra without distorting the stiff icosahedra [9,10]. X-ray Raman spectroscopy of the pressure evolution of the boron K-edge up to 30 GPa were performed on both pristine and shock recovered samples of B_4_C and showed no evidence of any valence transitions in the bridging boron atom [11] (the C-B-C bond is expected to yield at the point of molecular inversion.

It has also been suggested that the amorphization bands result from a phase separation which is triggered by anisotropic stresses. There has been an effort to minimize this effect by including light element dopants in B_4_C. A recent study suggested that silicon-doping of boron carbide (up to a maximum of 10%) increases the amorphization pressure from 25 to 55 GPa [12]. This opens the door to optimize and enhance the properties of boron carbide and widen its application potential.

We contribute to this growing body of experimental results with this report of measurements and analysis of the *P-V-T* data of B_4_C from ambient to 50 GPa and temperatures of the order of 2500 K using laser-heated DAC synchrotron powder X-ray diffraction techniques.

## 2. Experimental details

### (a) P-V-T equation of state

Two sets of data were collected at the beamline 16ID-B of HPCAT. In one run, B_4_C powder (CERECOM hot pressed powder supplied by US Army Research Laboratory. For details, see the report by Dandekar [5]) was loaded into a symmetric DAC with neon as medium and ruby as pressure sensor. Powder XRD data were collected using a focused, monochromatic, undulator beam at 0.6199 Å (20 keV) with a focal spot size of nominally 15–20 µm (90% width) and 5–7 µm FWHM. The tight focusing helped discriminate against the tungsten gasket (we preferred to use tungsten as gasket material to clearly delineate gasket diffraction that can interfere with the weak sample diffraction) and allowed us to obtain good powder patterns (the crystallite size was estimated to be of the order of 10 µm). The use of a cBN backing plate on the downstream side of the DAC allowed for a large X-ray aperture, needed for collecting a large solid angle. The reason we chose this low energy was to extract the first three diffraction peaks of B_4_C outside the shadow of the laser heating mirrors that were used in the high-temperature experiments. X-ray diffraction was recorded on a MARCCD.

**Figure 1.**
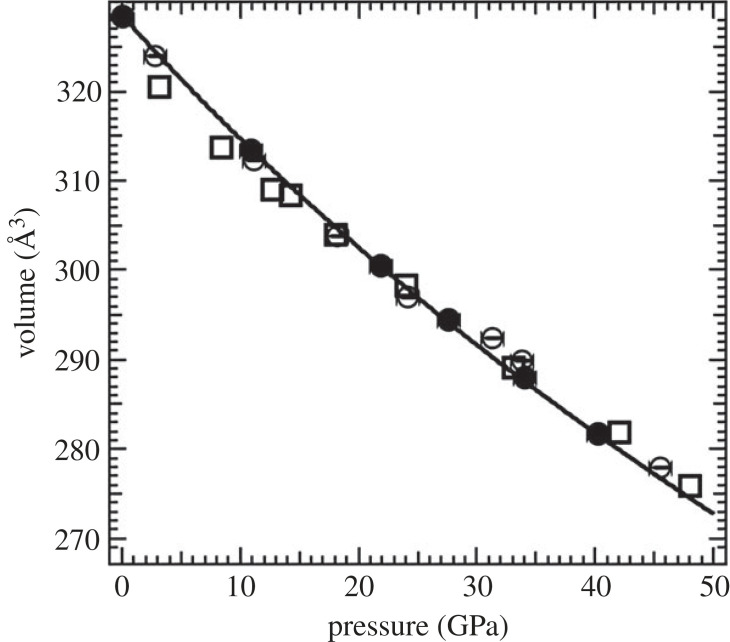
Pressure–volume data of B_4_C obtained from two different runs together with the fit to a second-order Birch–Murnaghan equation of state (solid line, *V*_0_ = 328.5 Å^3^, *K*_0_ = 221 GPa and d*K*_0_/d*P* = 3.3). The filled circles correspond to results from samples quenched from above 2000 K to ambient temperature which were used to constrain the fit. The empty circles represent the ambient data obtained without any thermal annealing. Both the filled and empty circles represent data obtained from an NaCl medium with MgO as pressure standard. The error bars in pressure represent the difference between pressure estimated from NaCl and MgO [13] diffraction data. Superposed are the P-V data obtained with a neon pressure medium where the pressure was determined using ruby fluorescence (empty squares).

In the second cell used for these experiments, a mixture of B_4_C and MgO (powder freshly prepared by crushing a single crystal that was then mixed with B_4_C powder in a diammoniate mortar and pestle under ethyl alcohol) was compacted into a pellet that was sandwiched between anhydrous NaCl plates. We used roughly 10% MgO by weight in the mixture. This sample was intended for performing the *P-V-T* measurements but also served as a reference for room temperature measurements especially when the high-temperature excursions helped relieve the stresses and sharpened the diffraction peaks. Additionally, *in situ* pressure standards NaCl and MgO could be used to obtain pressure. The equation of state presented in figure 1 was obtained from a combination of the two datasets.

**Figure 2.**
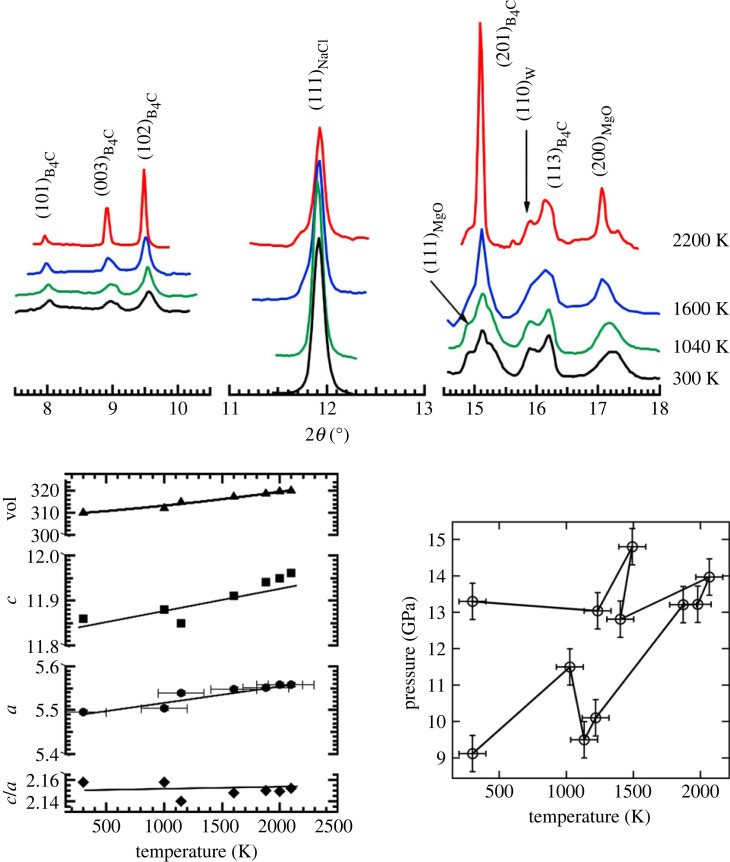
The top panel shows representative X-ray diffraction patterns obtained at a starting pressure of 9.1 GPa and subsequent laser heating. The diffraction peaks (111)_MgO_ and (200)_MgO_ were used to obtain the unit cell volume of MgO. The resulting pressures obtained from MgO P-V-T EOS [13] at various temperatures are plotted in the lower right panel and the cell constants of B_4_C obtained in the lower left panel. The error bars in pressure are estimated from the width of the MgO diffraction peaks while the error bars in temperature are deduced from the overall drift in temperature and the imbalance between the two sides (upstream and downstream heating spots). Since this was the first heating cycle, there was considerable drift in sample pressure. Subsequent heating at higher starting pressures did not show this large a drift and typically remained within approximately 2 GPa. (Online version in colour.)

The data at high temperatures were obtained on the laser heating diamond anvil cell (LHDAC) system of 16ID-B. Details of the system including temperature measurement and calibration, assuring coincidence of heating spot and probing X-ray beam, and varying the heating spot size, are reported elsewhere [14]. The LHDAC system allows one to modify the heating spot size and accordingly, the whole sample (approx. 50 µm in diameter) could be heated while the focused X-ray beam interrogated the central part of the sample. The efficient water-cooling jacket around the DAC assured that the sample did not drift in position while being heated (to within ±5 µm as estimated visually). Accordingly, diffraction patterns were obtained as the laser power was increased and concomitant spectra-radiometric data were obtained. The data acquisition program then saves the time record of temperatures, sample positions, filenames associated with temperatures and diffraction to allow assigning a temperature (or number of temperatures) with each diffraction; several temperatures could be recorded to estimate the overall drift in temperatures. Emission spectra were obtained from both sides of the sample using independent optical trains [14]. B_4_C is a weak scatterer and typical exposure times were 60 s while reliable emission spectra could be obtained in 10 s at low temperatures (less than 1500 K) and 1 s at temperatures above 2000 K. Figure 2*a* shows some representative diffraction patterns obtained in one such heating cycle. Figure 2*b* shows the estimated pressure from the cell volume of MgO. The P-V-T equation of state of MgO and the Mie–Grüneisen–Debye fit to the data as reported by Tange *et al.* [13] were used for this analysis.

The choice of MgO as an *in situ* pressure calibrant was dictated by the prior observation that B_4_C reacts with either Pt and/or Au and forms an inclusion compound at high temperatures. This irreversible process modifies the volume of the sample. A close inspection of the MgO diffraction peaks in figure 2*a* shows that the thermal expansion is clearly evident and, coupled with the fact that this is a powder mixture in which the only absorber of the IR laser is B_4_C, assures us that, (a) the mixture is homogenous and (b) that the two components have comparable grain sizes. MgO was hence used to obtain the thermal pressure at high temperatures. A total of nine starting pressures in the range of 8–50 GPa were chosen, and diffraction data were collected between ambient and 2500 K. Higher temperatures were, of course, easier to access at higher pressures but even at the lowest pressure of 8 GPa, the NaCl thermal buffer proved sufficient to stabilize temperatures of the order of 2200 K over extended periods of time. The one reason for this could be that the NaCl plates were obtained from an anhydrous sample that was kept inside an argon glove box and loaded into the DAC inside the glove box. After the NaCl plates were positioned inside the gasket chamber and on the opposing anvil face, the small sample of B_4_C + MgO was positioned in the gasket hole and the cell closed. The sample loading process therefore minimized the presence of any moisture that is usually detrimental to a stable laser heating especially at low pressures.

## 3. Results and analysis

There have been several studies that have reported the *P-V* equation of state of B_4_C at ambient temperature. These include experimental, DAC measurements [10,15] and DFT calculations [16]. More recently, there have been theoretical and experimental studies that addressed the plethora of shock data available on B_4_C (see Zhang *et al.* [17] for an extensive report). While both the experimental studies report a decreasing axial ratio with increasing pressure, the single crystal study of Dera *et al.* [10], which followed an earlier neutron diffraction study [9], postulates an inverse molecular behaviour driven by the incompressible B_4_C icosahedra (two centred bonds) in comparison with the more compressible intra-icosahedra, C-B-C three centred bonds. Their single-crystal data indicate the onset of this transition to be around 10 GPa [10], which was consistent with the neutron diffraction studies reported earlier [9].

**Figure 3.**
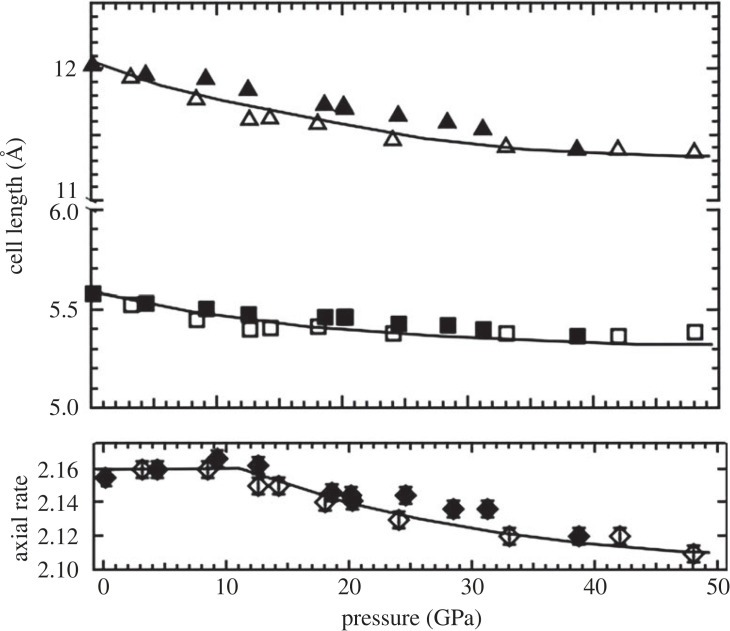
Unit cell lengths and axial ratio of hexagonal B_4_C obtained from the ambient temperature data both with MgO pressure marker and NaCl medium (solid symbols) and neon pressure medium and ruby pressure standard (open symbols). The lines are a guide to the eye and not any particular fits. While the cell lengths and volume (figure 1) show a monotonic decrease with increasing pressure, the axial ratio shows three distinct regions with a nearly monotonic decrease in the pressure interval 10–30 GPa. The solid symbols also include data obtained before and after annealing at high temperatures.

We first discuss the room temperature *P-V* equation of state. As indicated earlier, two sets of data were collated into one fit (figure 1). A third-order Birch–Murnaghan equation of state was fitted to all the data and compared with only the data obtained from a similar quasihydrostatic media. In addition, both *V*_0_ and (d*K*/d*P*)_0_ were varied in the least-squares fit carried out with the help of the software EosFIT [18]. The *V*_0_ obtained from the fit was found to be within 2*σ* of the refined cell constants of the starting material on a D3 phaser. This was needed to assure ourselves of the ability to cross compare our EOS values with earlier published data whose zero pressure volumes were different and could reflect different starting stoichiometries [2]. In addition to fitting all the P-V data, we chose to fit a subset of the results with MgO as the pressure standard. These data obtained from samples after quenching from high temperatures correspond to those that have been heated sufficiently to release non-hydrostatic stresses. We fixed *V*_0_ as indicated above to 328.4 Å^3^ and varied *K*_0_ and (d*K*/d*P*)_0_ to obtain values of 221(2) GPa and 3.3(5), respectively. When all the MgO data were used, we obtained values of 204(14) GPa and 4.4(11), respectively. This variation reflects the effect of non-hydrostatic stresses that increases the scatter of the data (figure 1). In fact, our neon data in which ruby was used to determine pressure also show a systematic deviation above 10 GPa and probably represents a similar effect as sample bridging by the anvils, since the ruby R_1_−R_2_ doublet remained well resolved up to at least 30 GPa. While the values of *V*_o_ are similar, the bulk modulus derived from single-crystal X-ray diffraction [10], powder X-ray diffraction [15] and neutron diffraction [9] vary in the range 200–300 GPa; this variation has been attributed to varying starting stoichiometries and stress state of the sample even during quasi-hydrostatic compression studies [2,17,19]. We also concur with earlier studies regarding the axial ratio in the lower pressure range. In contrast to earlier studies, however, we find that the axial ratio, which starts falling around 10 GPa, recovers and stiffens above 30 GPa. This behaviour is not reflected in any significant way by the cell lengths that show a monotonic pressure dependence (figure 3).

In the Mie–Grüneisen formulation, the EOS of solids is generally represented as

3.1 $$P(V,T)=P_{T_{0}}(V)+\Delta P_{\text{th}}(V,T).$$

Here, *P*_To_(*V*) is the 300 K pressure that is related to the volume *V* through the third-order Birch–Murnaghan equation,

3.2 $$P_{T_{0}}=\frac{3}{2}K_{T_{0}}\left[{\left(\frac{V}{V_{0}}\right)}^{-7/3}-{\left(\frac{V}{V_{0}}\right)}^{-5/3}\right]\left\{1+\frac{3}{4}{K^{\prime }}_{T_{0}}\left({\left(\frac{V}{V_{0}}\right)}^{-2/3}-1\right)\right\}.$$

Here, *P*_T0_ is the pressure at a reference temperature, which we assume is 300 K. The factors *V*_0_, *K*_T0_ and K_T0_^′^ are obtained from the 300 K *P-V* EOS fit. The thermal pressure Δ*P*_th_, is expressed in terms of the difference of internal thermal energies Δ*E*_th_ between *T*_o_ and *T* as

3.3 $$\Delta P_{\text{th}}(V,T)=\frac{\gamma }{V}\Delta E_{\text{th}}(V,T)=\frac{\gamma }{V}[E_{\text{th}}(V,T)-E_{\text{th}}(V,T_{0})],$$

where *γ* is the Grüneisen parameter. In the Debye model, the internal energy at a given temperature is given by the integral

3.4 $$E_{\text{th}}(V,T)=9nRT{\left(\frac{\Theta_{D}}{T}\right)}^{-3}\int_{0}^{\Theta_{D}/T}\frac{x^{3}}{e^{x}-1}\text{d}x,$$

where *R* is the gas constant, *n* is the number of atoms per formula unit (5 for B_4_C) and *θ*_D_ is the Debye temperature. The Grüneisen parameter represents the volume dependence of *θ*_D_ and can be expressed as

3.5 $$\gamma (V)=\gamma_{0}{\left(\frac{V}{V_{0}}\right)}^{q},$$

with

3.6 $$\Theta_{D}(V)=\Theta_{0}\;\exp\left[-\frac{\gamma (V)-\gamma_{0}}{q}\right].$$

Thus, the thermal pressure can be calculated with three parameters (*θ*_0_, *γ*_0_ and *q*), assuming *q* is a constant [20,21]. Tange *et al.* [13] propose a model where the volume dependence of *γ* is expressed in the form

**Figure 4.**
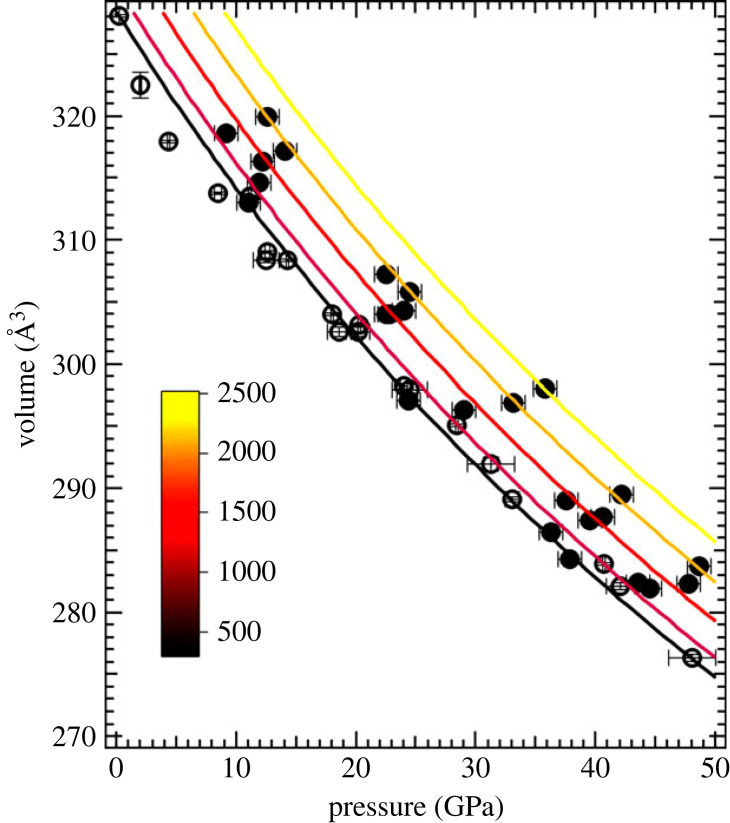
Results of the Mie–Grüneisen–Debye EoS of the P-V-T data of B_4_C. In the *χ*^2^ minimization, the parameters *V*_0_, *K*_0_ and d*K*_0_/d*P* were held fixed using the 300 K values while *γ*_0_ = 0.8 and *θ*_0_ = 1425 were fixed to obtain a best fit for *q* = 2.1 (see table 1 for full details). (Online version in colour.)

3.7 $$\gamma (V)=\gamma_{0}\left\{1+a\left[{\left(\frac{V}{V_{0}}\right)}^{b}-1\right]\right\},$$

with two adjustable parameters *a* and *b* that extrapolates to *γ* = *γ*_0_ when *a* = *b* = 0 and *γ* = *γ*_0_(*V*/*V*_0_) when *a* = *b* = 1 (*γ*/*V* is a constant, a model traditionally used in shock compression analysis [19]). The thermal pressure is hence calculated using the four parameters (*θ*_0,_ *γ*_0_, *a* and *b*). The high degree of correlation between the four coefficients forced us to choose a fitting with *a* = 1 and *b* varied. This represents a situation where only one refine-able parameter *q* is used. In the same table, the values of *γ* and *θ*_D_ reported from static measurements [22] and dynamic measurements [19] are also shown for comparison. The results of our fit are summarized in figure 4 and table 1. We fixed the ambient pressure parameters *γ*_0_ and *θ*_0_ obtained from ambient pressure measurements [2,22,26] and refined the parameter *q*. For details about the error analysis and the mathematical details of the fitting algorithm, see the attached supplementary materials. The data used in the fit are also listed in the electronic supplementary material [23].

**Table 1.** Parameters from the MGD fit of the *P-V-T* data of B_4_C. The full data in the pressure range 8–50 GPa and 300–2500 K were fitted using a weighted chi-squared minimization (for details, see the electronic supplementary material [23]). The result of this fit is displayed in figure 4.

	our results	static results	dynamic results
*V*_0_ (Å^3^)	328.4 (fixed)		
*K*_0_ (GPa)	221 (fixed)		
(d*K*/d*P*)_0_	3.3 (fixed)		
*θ*_0_ (^0^K)	1450 (fixed)	1470 [24]	
		1520 [25]	
*γ*_0_	0.8 (fixed)	0.8 [22]	0.4(3) [19]
			0.9(2) [5]
*a*	1.0 (fixed)		
*b*	2.1 (varied)		

**Figure 5.**
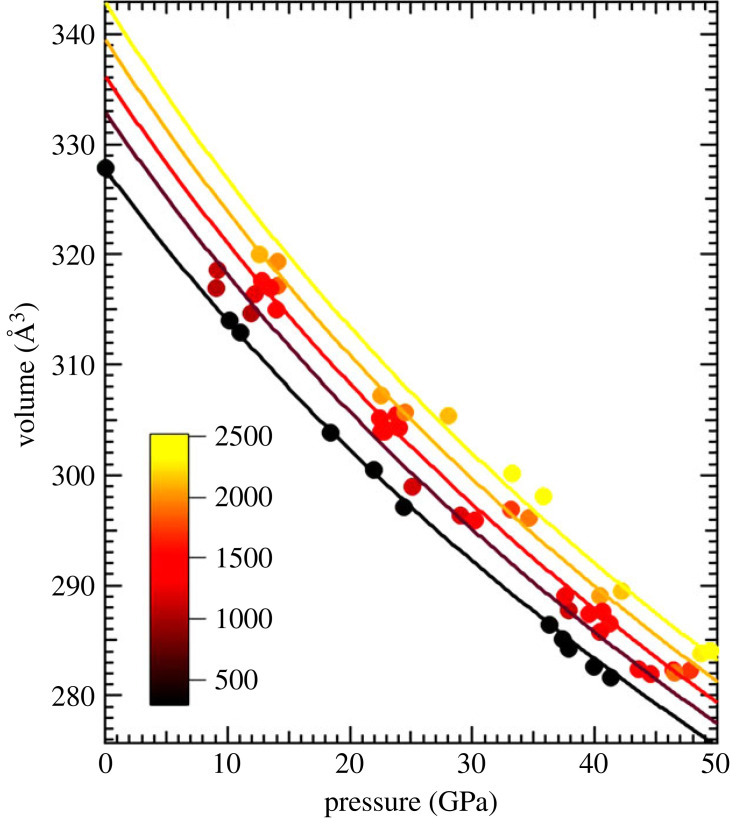
Results of the Berman fit [28] of the P-V-T data using EosFit7c [29]. The thermal expansion parameters and d*K*_0_/d*T* were varied while the 300 K parameters for *V*_0_, *K*_0_ and *K*_0_′ were held fixed to the values listed in table 1 and depicted in figure 1. The data displayed here are clubbed into different isotherms assuming a spread of ±250 K for ease of representation while the analysis was performed with the relevant pressures (as derived from MgO) and temperatures as obtained from the radiometry weighted with their respective standard deviations. (Online version in colour.)

A parametrized conventional isothermal EOS fit was also made by assuming an ambient pressure thermal expansion parameter *α*, and a temperature dependence of the bulk modulus d*K*/d*T* [27]. Although such a fit is unphysical (especially in cases where the d*K*/d*T* contribution can change drastically due to some elastic relaxation, as is certainly expected in B_4_C and materials where d*K*/d*P* can deviate appreciably from an empirical value of 4, again shown by B_4_C). The ease of such a parametric description of the *P-V-T* behaviour lies in the fact that a ready estimate of K(P, T) can be made as input into other measurements such as Brillouin scattering or ultrasonic measurements for sound velocity measurements. These parameters are listed in table 2 and the fit is summarized in figure 5. The fit to the whole dataset was performed using EoSFit7c [18].

**Table 2.** Parameters from the Berman fit [28] to the *P-V-T* data using EoSFit7c [29]. The fitting was performed by varying only the parameter *α*_o_ in equation (3.9) and the cross term d*K*_0_/d*T*. The result of this fit is displayed in figure 5.

*V*_0_ (Å^3^)	328.4 (fixed)
*K*_0_ (GPa)	221 (fixed)
*K_p_*	3.3 (fixed)
*K_pp_*	−0.0166 (implied value)
d*K*_0_/d*T*	−0.008(3)
*α*_o_ (K^−1)^	1.94(16) × 10^5^
*α*_1_ (K^−2^)	0.0573 × 10^8^ (fixed)

The same third-order Birch–Murnaghan EOS was used to describe the behaviour of *P*(*V, T*), where *V*_0_ in equation (3.2) was replaced by *V*_o_(*T*) as given by

3.8 $$V_{o}(T)=V_{0}\exp\int_{T_{0}}^{T}\alpha (T)\;\text{d}T.$$

Where the ambient pressure thermal expansion coefficient is assumed to be linear,

3.9 $$\alpha (T)=\alpha_{0}+\alpha_{1}(T),$$

and the thermal variation of the bulk modulus is represented by a cross term d*K*_0_/d*T*. The parameters *α*_0_, *α*_1_ and d*K*_0_/d*T* are free parameters that can be varied for the fit of the *P-V-T* data. In our analysis, however, we fixed *α*_1_(*T*) using the ambient pressure thermal expansion measurements of B_4_C reported elsewhere [26] and refined *α*_0_ since the two parameters are observed to be highly correlated. In all cases, we weighted the observed variables *P, V, T* by their statistical variances also reported in the SM.

In conclusion, we present *P-V-T* data for laser-heated B_4_C in the pressure 0.1–50 GPa range and temperatures between 1000 and 2500 K. The use of MgO as the *in situ* pressure sensor made it possible to estimate thermal pressure and the data obtained could be fit with a Berman *P-V-T* equation of state as well as a Mie–Grüneisen–Debye thermal model. In variance with several dynamic pressure studies, B_4_C remains structurally stable in this *P-T* range, only displaying the previously reported relaxation at 10 GPa (and at ambient temperature) which is accompanied by a monotonic reduction of the axial ratio. This is best exemplified by the fact that only a single *P-V-T* equation of state was capable of describing the whole dataset. The Grüneissen parameter *γ*_0_ and the volume-dependent parameter *γ*(*V*) indicate that the dominant reference modes prevalent are those related to the icosahedra [30], unlike the reports from shock measurements of *γ* [19].

## Data Availability

The data are provided in the electronic supplementary material [23].
